# Effectiveness of Cognitive Training for School-Aged Children and Adolescents With Attention Deficit/Hyperactivity Disorder: A Systematic Review

**DOI:** 10.3389/fpsyg.2019.02983

**Published:** 2020-01-14

**Authors:** Andreia Veloso, Selene G. Vicente, Marisa G. Filipe

**Affiliations:** Faculty of Psychology and Education Sciences, Centre for Psychology, University of Porto, Porto, Portugal

**Keywords:** attention deficit/hyperactivity disorder, ADHD, cognitive training, executive functions, intervention, review

## Abstract

Problems with executive functions (EF) are hallmark characteristics of Attention Deficit/Hyperactivity Disorder (ADHD). Therefore, this review analyzed the efficacy of cognitive training for EF in reducing ADHD symptomatology and improving educational, interpersonal, and occupational outcomes in children and adolescents with this disorder. A systematic search, using a PICO (population/participant, intervention/indicator, comparator/control, outcome) framework was carried out. From 2008 to 2018, resorting to EBSCO*host*, the following databases were searched: Academic Search Complete, ERIC, MEDLINE with Full Text, PsycARTICLES, PsycINFO, and Psychology and Behavioral Sciences Collection. Twenty-two studies were included in this review. Of the 18 studies that reported performance-based measures of EF, 13 found improvements and five did not. Overall, 17 studies showed positive transfer effects on ADHD symptomatology, EF, academic improvement, reduced off-task behavior, and/or enhanced social skills. Of the nine studies that performed follow-up sessions, seven concluded that the treatment effects were maintained over time. In sum, results showed that cognitive training can be an effective intervention for children and adolescents with ADHD and might be a complementary treatment option for this disorder.

Attention Deficit/Hyperactivity Disorder (ADHD) is a neurodevelopmental disorder marked by persistent symptoms of inattention, hyperactivity, and/or impulsivity (American Psychiatric Association, 2013). Children diagnosed with this disorder present difficulties in the ability to pay attention, restrain movements, inhibit impulses, and regulate behavior (Roberts et al., 2015) that affect communication, daily living, and socialization (Weyandt and Gudmundsdottir, 2015). Importantly, according to the literature, these difficulties arise from deficits in executive functioning (e.g., Rapport et al., 2001; Sonuga-Barke, 2003; Willcutt et al., 2005; Nigg, 2006; Barkley, 2015).

Even though there is no agreed-upon definition, executive functions (EF) can be viewed as a multidimensional construct that encapsulates higher-order cognitive processes responsible for guiding, directing, and managing cognitive, emotional, and behavioral functions, particularly during novel problem situations (Gioia et al., 2000). There are several cognitive processes associated with EF, but the major elements include anticipation, goal selection, planning, initiation, self-regulation, mental flexibility, attention, and utilization of feedback (Anderson, 2002). These processes develop throughout childhood and adolescence and are invaluable to the cognitive, behavioral, emotional, and social functioning of the individual (Anderson, 2002), originating a variety of difficulties when impaired (Brown, 2013).

Consequently, EF has been broadly investigated in individuals with ADHD and, even though results have been incongruous, studies showed poor performance of children with ADHD on EF tasks when compared to typically developing peers (e.g., Nigg et al., 2002; Willcutt et al., 2005). For instance, a meta-analytic review composed of 83 studies evaluated the validity of the EF theory in this population and observed that children with ADHD display significant deficits in inhibitory control, vigilance, working memory, and planning (Willcutt et al., 2005). A few studies also found difficulties in processing speed (Nigg et al., 2002; Lawrence et al., 2004; Pasini et al., 2007; Yáñez-Téllez et al., 2012), cognitive flexibility (Lawrence et al., 2004; Geurts et al., 2005; Yáñez-Téllez et al., 2012), and sustained attention (Nigg et al., 2002; Trani et al., 2011; Yáñez-Téllez et al., 2012). Thus, problems with EF seem to be hallmark characteristics of this disorder.

Treatment options for ADHD are limited and, frequently, involve the prescription of psychostimulant medication as a first line treatment (Yildiz et al., 2011). Improvements in behavior, attention, interpersonal interactions, cognition (Biederman and Spencer, 2008), and EF (e.g., Barnett et al., 2001; Swanson et al., 2011) reinforce the short-term efficacy of psychostimulant medication, of which methylphenidate and dextroamphetamine are the most prescribed (Rabipour and Raz, 2012). Nevertheless, the limitations of such medication (e.g., short-term effects, unknown long-term effects, side-effects such as insomnia and lack of appetite) prompt parents and professionals to look for another treatment options (Rabipour and Raz, 2012). Thus, efforts have been made to develop non-pharmacological interventions that decrease ADHD symptomatology, and cognitive training has been considered a potential intervention. As reported by Vinogradov et al. (2012), due to brain plasticity, cognitive training can strengthen and develop essential brain networks and underlying cognitive processes by exposing the brain to well-defined learning tasks, resulting in more adaptive behaviors across contexts.

The tasks presented in cognitive training interventions vary extensively, are usually conferred as games, and can be presented through a computer or pen and paper format, aiming to improve a plethora of abilities such as working memory, attention, inhibitory control, planning, and cognitive flexibility. As it is important to keep the child engaged, motivated, and practicing at a level that is in accordance with or slightly above their current abilities (Diamond, 2012; Rapport et al., 2013), this training is usually adaptive (i.e., the difficulty of the task is adjusted to the performance of the child; Rapport et al., 2013). Thus, it has been proposed that cognitive training can reduce ADHD symptomatology and improve functioning by addressing the neuropsychological deficits thought to mediate its pathophysiology (Cortese et al., 2015).

A meta-analytic review carried out by Cortese et al. (2015) examined the effects of cognitive training on ADHD symptomatology, neuropsychological deficits, and academic skills in children and adolescents with ADHD. The authors concluded that there were significant effects of training on ADHD symptoms when considering unblinded raters. Yet, these results were drastically reduced when analyses were limited to trials with active control groups or where raters were blind to treatment conditions. Additionally, significant performance improvements in objective measures of visual and verbal working memory were reported, while there were no effects on inhibition or attention. However, these effects of training on working memory did not extend to academic outcomes. Other reviews found similar results (e.g., Karch et al., 2013; Rapport et al., 2013; Sonuga-Barke et al., 2013). However, the majority of the studies included in these previous reviews focused on a single EF, such as working memory, inhibitory, and attention (or a combination of these). Given that children with ADHD display a diverse set of EF deficits, and training multiple EF might be a more effective strategy than focusing on a single EF domain (Dovis et al., 2015), a systematic literature review focusing on the training of multiple EF domains is lacking.

As it has been proposed that cognitive training for improving executive functioning can reduce ADHD symptomatology (e.g., Cortese et al., 2015), our main aim is to update and extend the findings of previous systematic reviews and meta-analyses, characterizing the current literature on cognitive training interventions for EF in children and adolescents diagnosed with ADHD. We, thus, examine the outcomes within the included studies to determine the efficacy of cognitive training assessed by performance-based measures of EF and behavioral/EF questionnaires. Additionally, we intended to evaluate the transfer effects (i.e., generalization of training effects to other non-trained tasks) and possible maintenance of gains reported in each study. As such, we developed the following research question using the PICO framework: In children and adolescents with ADHD, is cognitive training for EF, compared to other types of intervention, typically developing controls, or placebo, effective in developing executive functioning, reducing symptomatology, and improving educational, interpersonal and/or occupational outcomes? (cf. Table 1).

**Table 1:** PICO (population/participant, intervention/indicator, comparator/control, outcome) framework.

**PICO framework**	
Population	Children and adolescents with ADHD, aged 3–14 years old
Intervention	Cognitive training of at least one domain of executive functioning
Comparison	Other types of intervention and/or a placebo condition and/or healthy controls
Outcome	EF, ADHD symptomatology, and/or functional outcomes (i.e., educational, interpersonal, and/or occupational)

## Methods

### Search Strategy

**Figure 1:**
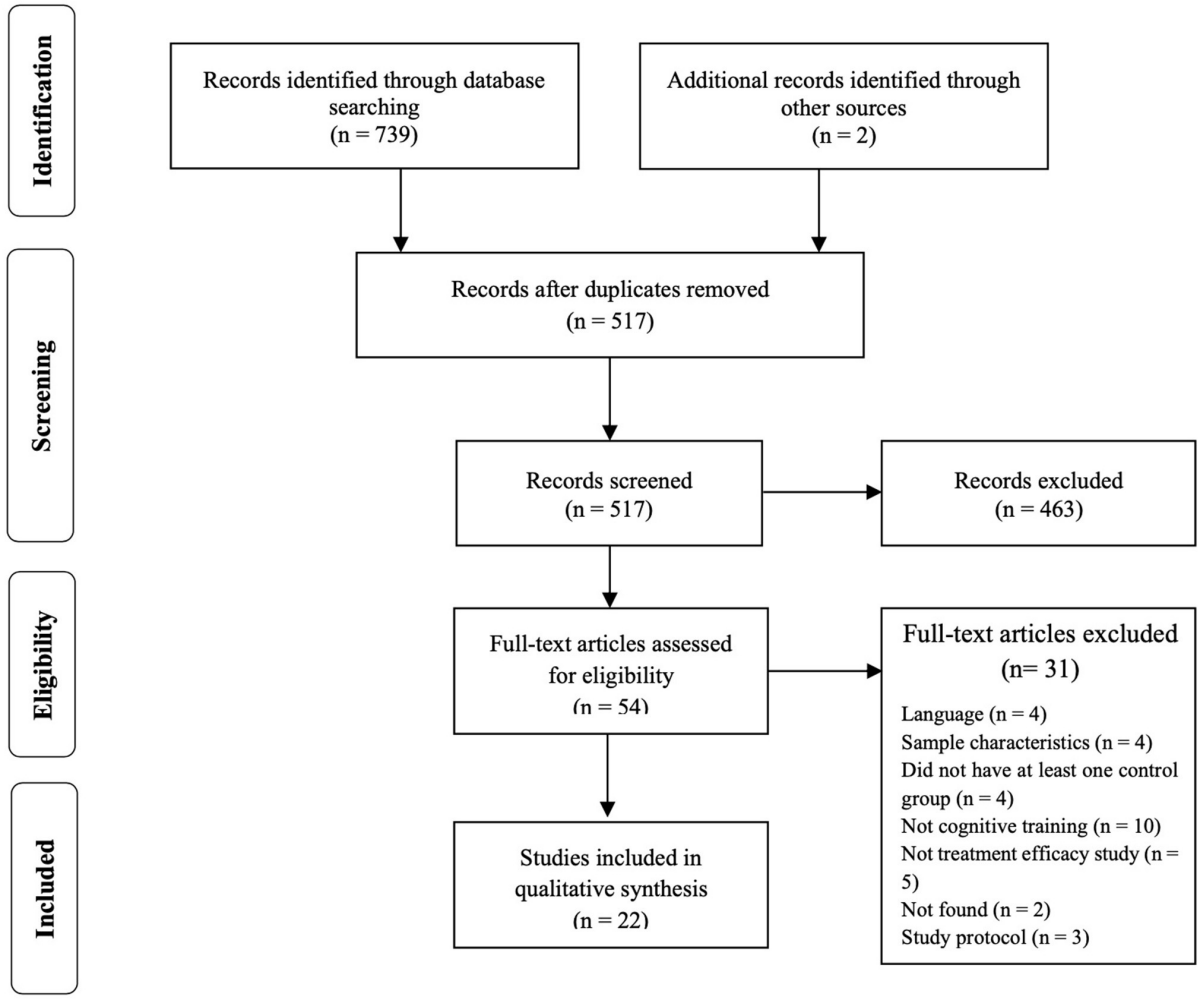
Preferred Reporting Items in Systematic Reviews and Meta-Analyses (PRISMA) flow diagram of selection of studies.

The systematic literature search was conducted between January and February 2018, resorting to EBSCO*host* (Academic Search Complete, ERIC, MEDLINE with Full Text, PsycARTICLES, PsycINFO and Psychology and Behavioral Sciences Collection). The search keywords were operationalized using a Population/Participant, Intervention/Indicator, Comparator/Control, Outcome(s) (PICO) search framework (cf. Table 1). Focusing on the last 10 years (2008–2018), the keywords executive function^*^ **OR** *executive functioning* **AND** *cognitive training* **OR** intervention^*^ **AND** *Attention Deficit Hyperactivity Disorder* **OR** *ADHD* were used to conduct the search. Every reference was, then, conveyed to Mendeley and Rayyan, a website developed to assist systematic review authors to perform study selection (Ouzzani et al., 2016), and duplicates were removed. Every study was initially identified by title and abstract, according to the inclusion criteria. Figure 1 details the process of study selection.

### Inclusion and Exclusion Criteria

In order to be included in this review, studies had to: (1) include children with ADHD aged 3–14 years; (2) include a cognitive intervention/training focusing on one or more components of EF; (3) include an active and/or a passive control condition; (4) include measures of EF, ADHD symptomatology, academic achievement and/or interpersonal relationship quality as outcomes; and (5) be published in English.

Reasons for exclusion entailed: (1) reviews, meta-analysis, dissertations, book chapters, and study protocols; (2) studies involving different intervention technics (e.g., mindfulness, neurofeedback); (3) studies focused on different neurodevelopmental disorders (e.g., learning difficulties, Autism Spectrum Disorder) or clinical groups (e.g., Neurofibromatosis); (4) studies including different age ranges (e.g., adults); (5) studies that do not include at least one outcome measure of EF, ADHD symptomatology, academic achievement and/or interpersonal relationship quality; and (6), papers not published in English.

Studies were not excluded if children presented comorbid diagnoses or maintained the course of pharmacological treatment during interventions.

After screening each study by title and abstract, the full texts were analyzed and included if they fulfilled the stipulated criteria. Two additional studies (Green et al., 2012; Johnstone et al., 2012) were hand-searched and included in this review.

### Risk of Bias (Quality) Assessment

**Figure 2:**
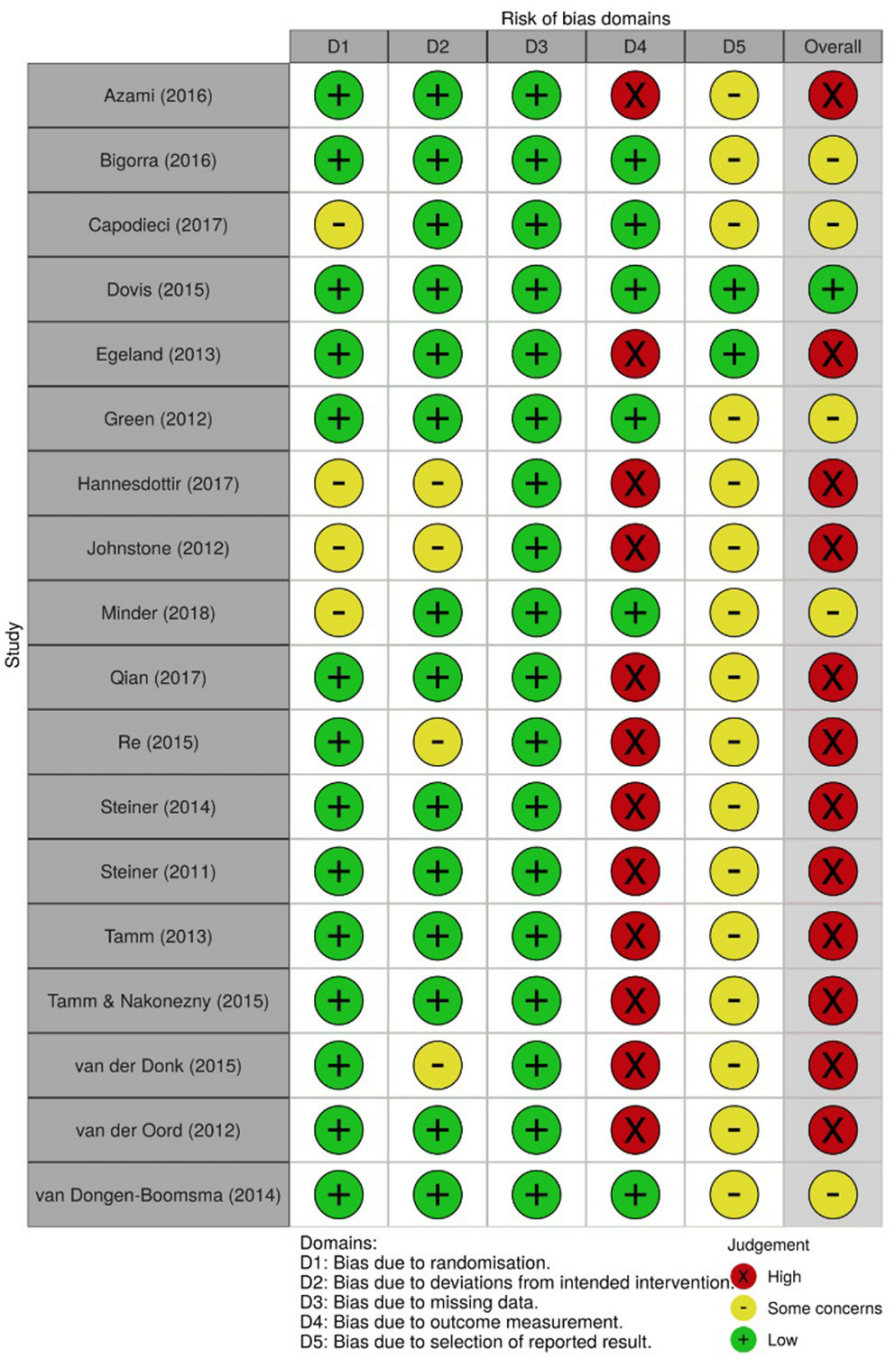
Risk of bias summary for all randomized trials included.

**Figure 3:**
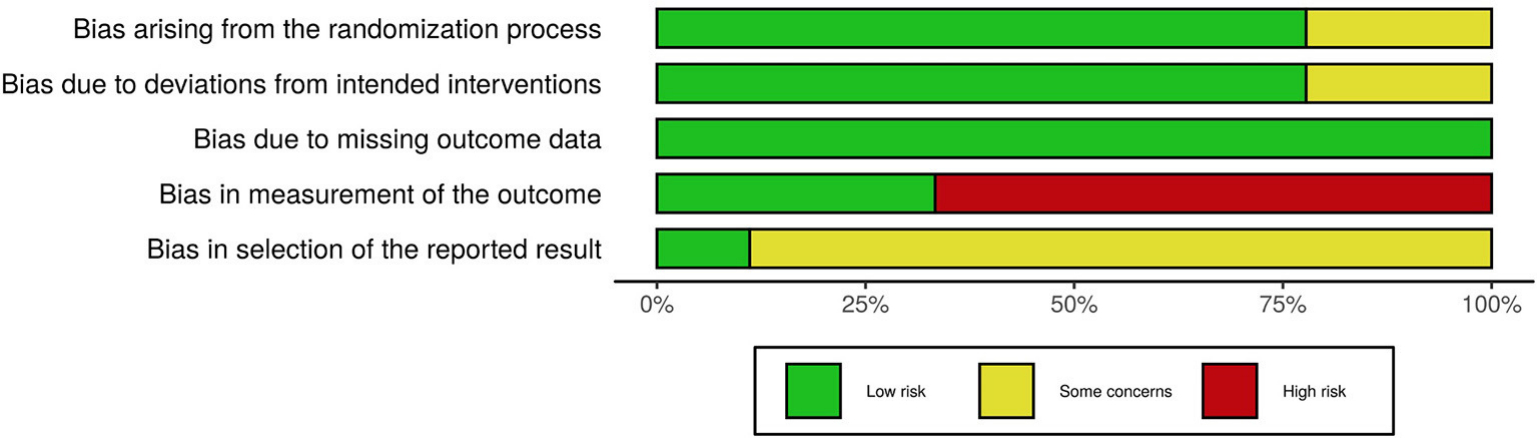
Risk of bias graph for all randomized trials included.

**Figure 4:**
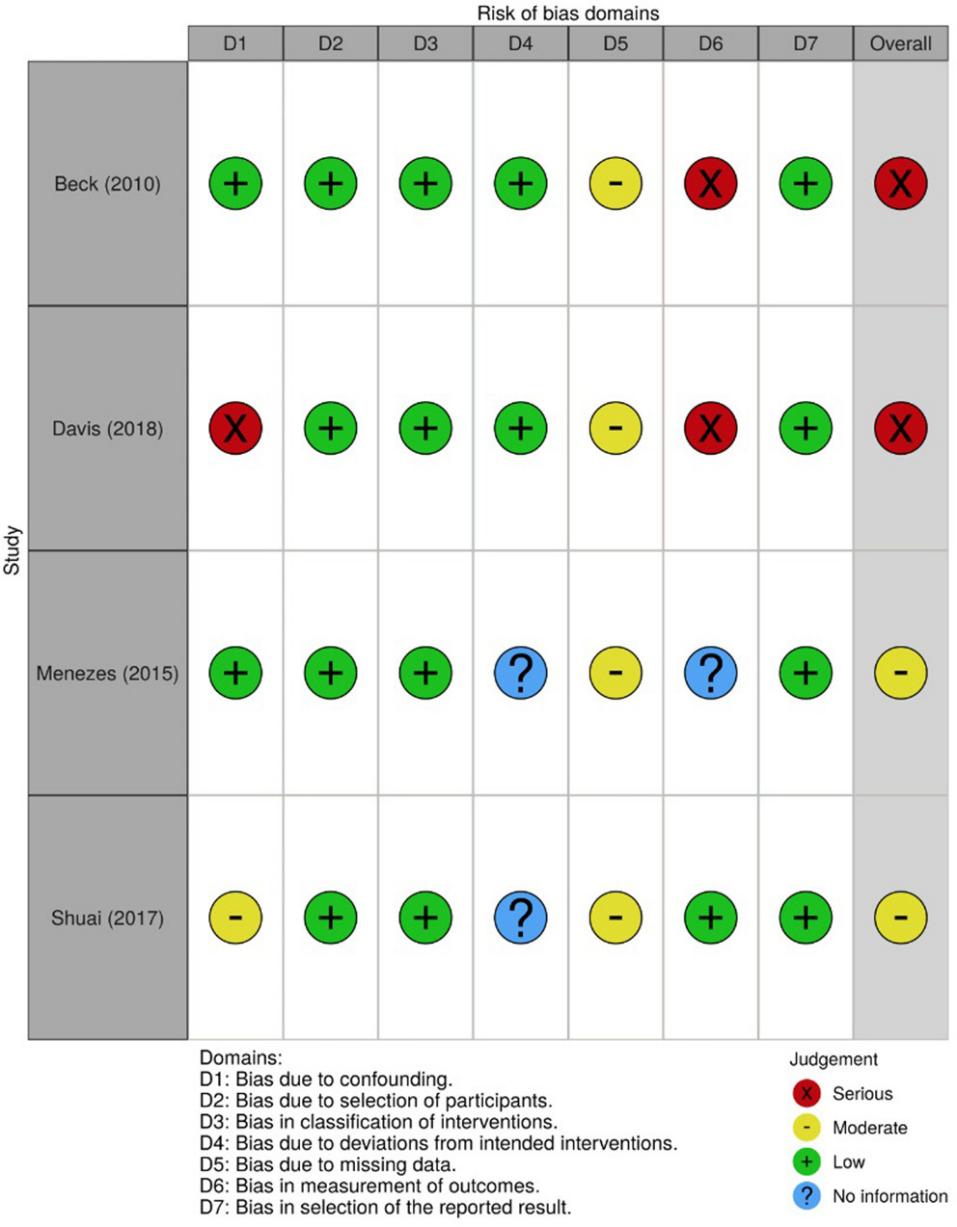
Risk of bias summary for all non-randomized trials included.

**Figure 5:**
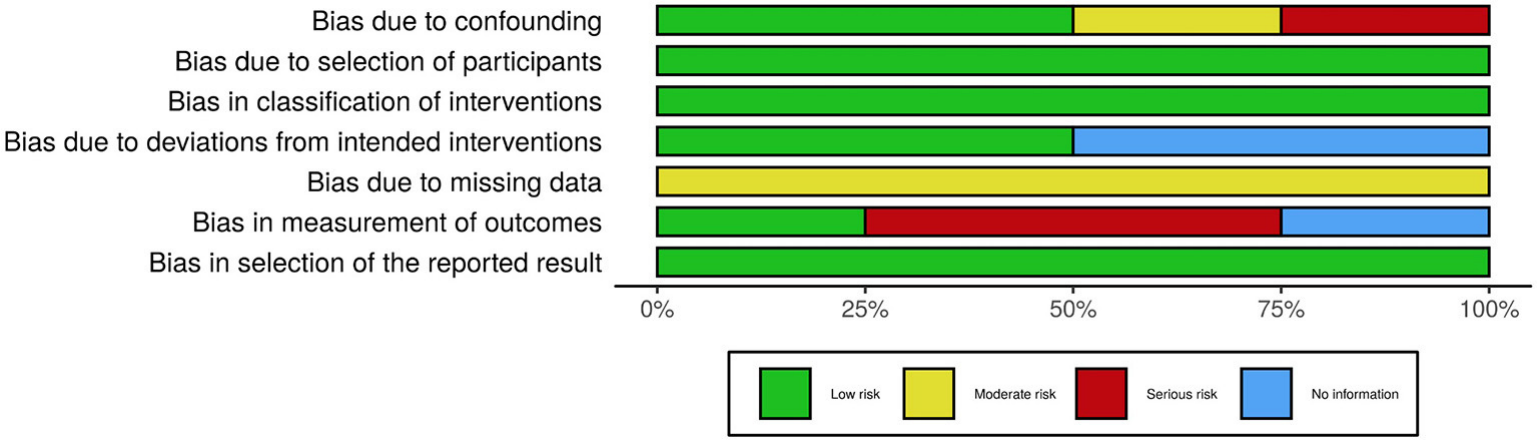
Risk of bias graph for all non-randomized trials included.

In order to assess the risk of bias of the included studies, the Cochrane Collaboration tools were used, namely the RoB 2.0 (Sterne et al., 2019) for the randomized trials and the ROBINS-I (Sterne et al., 2016) for the non-randomized trials. The RoB 2.0 assesses five domains of bias, specifically: (1) bias due to randomization, (2) bias due to deviations from intended intervention, (3) bias due to missing data, (4) bias due to outcome measurement, and (5) bias due to selection of reported result. The ROBINS-I, one the other hand, assesses: (1) bias due to confounding, (2) bias due to selection of participants, (3) bias in classification of interventions, (4) bias due to deviation from intended intervention, (5) bias due to missing data, (6) bias in measurement of outcomes, and (7) bias in selection of the reported result. Risk of bias was independently assessed by AV and MF and disagreements were resolved through discussion. Figures 2–5, designed with the *robvis* web app (McGuiness, 2019), depict the plots obtained from these analyses.

### Data Extraction

Data obtained from each study encompassed sample characteristics, study design, settings, intervention materials, outcome measures, and main findings.

## Results

### Trial Flow

A total of 739 articles were identified from the databases using the search strategy previously reported. Two additional references were hand-searched and included in this review. Two hundred and twenty-four duplicates were removed, and 517 articles were assessed by title and abstract. Of these, 463 reports were excluded since they did not fulfill the predetermined criteria. Hence, 54 papers were included and their full-text analyzed, of which 22 met inclusion criteria. Again, in Figure 1, the study selection process is presented in a PRISMA flow diagram (Moher et al., 2009).

### General Study Characteristics

The age range of the participants included in each study varied extensively and most of the studies analyzed children aged between 3 and 12 years old (cf. Table 2).

**Table 2:** Study and intervention characteristics.

**References**	**Country**	**Setting**	**Method**	**Age range**	**Comorbidities**	**Type of comparison groups**	**Comparison groups**	**Treatment (*n*)**	**Comparison groups (*n*)**	**Intervention**	**Duration | Intensity**	**Targets of intervention**	**Outcome measures**
Azami et al. (2016)	Iran	Clinical	RCT	7–12	–	1 Active1 Passive	Medication; Placebo	12	11 + 11	Persian software of working memory training; Lumosity; The Amazing Brain Train	20 × 90 min sessions (2 months)	Attention, inhibition, short-term memory, planning, processing speed	CPT, TOL, Digit Span, RCPM, Span Board, SNAP-IV
Beck et al. (2010)	USA	Home	CT	7–17	ODD; CD; Anxiety; Mood disorders	Passive	Waitlist	27	24	Cogmed RM	25 × 40 min sessions (6 weeks)	Verbal and visuo-spatial working memory	BRIEF, CRS, number of DSM–IV–TR inattentive symptoms
Bigorra et al. (2016)	Spain	Home	RCT	7–12	ODD; CD	Passive	Placebo	36	30	Robomemo^®^ Cogmed Working Memory Training™	25 × 45 min sessions (5 weeks)	Working memory	CRS, BRIEF, CBCL, TRF, SDQ, WFIRF, Digit Span, Letter-Number sequencing, Spatial Span, CPT, TOL, TMT, WCST
Capodieci et al. (2018)	Italy	School	RT	5	–	Passive	Non-training	38	36	Activities presented in the manual published by Caponi et al.	16 × 60 min sessions (8 weeks)	Working memory	IPDDAI; IPDDAG; Span Backward; Selective WM; Span Forward; Walk–No Walk Test; Matching Figures
Davis et al. (2018)	USA	Home	OL/OT	8–12	–	Passive	Non-ADHD	40	40	*Project:* EVO	27 × 45 min sessions (6 weeks)	Cognitive interference	Test of Variables of Attention, BRIEF, CANTAB
Dovis et al. (2015)	Netherlands	Home	RCT	8–12	–	1 Active1 Passive	Partially active condition; Placebo	31	28 + 30	Braingame Brian	25 × 35–50 min sessions (5 weeks)	Working memory, inhibition and cognitive flexibility	Stop task, Stroop test, CBTT, Digit Span, TMT, RCPM, BRIEF, DBDRS
Egeland et al. (2013)	Norway	School	RCT	10–12	–	Passive	Treatment as usual	33	34	Robomemo^®^ Cogmed Working Memory Training™	25 × 45 min sessions (5–7 weeks)	Working memory	Color Word (CW) and Trail Making (TMT) tests from the Delis-Kaplan Executive Function System, CPT-II, Children's Auditory Verbal Learning Test-2, Benton Visual Retention Test−5th edition, ADHD-Rating Scale IV, SDQ, BRIEF
Green et al. (2012)	USA	Home	RCT	7–14	Unclear	Passive	Placebo	12	14	WM Cogmed	25 × 40 min sessions	Working memory, off-task behavior	RAST, CRS, WM index (Digit-Span and Letter-Number Sequence from WISC-IV)
Hannesdottir et al. (2017)	Iceland	–	RT	8–10	Unclear	1 Passive1 Active	Waitlist; Parent group	16	14 + 11	OutSMARTers Program	10 × 2 h sessions (5 weeks)	Social skills, self-regulation, and executive functions	ADHD Rating Scale–IV, SSRS, Emotion Regulation Checklist, SDQ, Lumosity, WISC-IV subtests (Coding, Arithmetic, and Letter–number sequence)
Johnstone et al. (2012)	Australia	Home	RT	7–13	–	Passive	Waitlist	83	45	Feed the Monkey, Go Go No-Go	25 × 20 min sessions (4–5 weeks)	Working memory, inhibitory control	Visual Go-No Go task, Oddball task, Flanker task, Counting Span, Digit Span, CRS, purpose-designed Behavior Rating Scale
Menezes et al. (2015)	Brazil	Clinical	OL/OT	7–13	–	Passive	ADHD	8	10	PIAFEx	≈64 × 60 min sessions (8 months)	EF and self-regulation	Computerized Stroop Test, CAT, TMT, WCST, Auditory WM Test; Visual WM Test, FAS, Animals Verbal Fluency Test, CHEXI
Minder et al. (2018)	Switzerland	Clinical School	RCT	8–16	–	Active	Neurofeedback	39	38	CogniPlus	(5–14 weeks)	Attention, working memory, inhibition	CRS, BRIEF
Qian et al. (2017)	China	Clinical	RCT	6–12	ODD; LD; Phobias	2 Passive	Waitlist; Healthy control group	38	30+23	Based on Dawson and Guare's (2010) training of executive skills for children	12 × 60 min sessions (12 weeks)	Inhibition, planning, working memory, time management, sustained attention, organization, cognitive flexibility	Stroop Task, Rey-Osterrieth Complex Figure Test, TMT, BRIEF, ADHD-RS-IV, WFIRF
Re et al. (2015)	Italy	School	RT	5	–	Active	Non-training	26	13 + 13	Development of Concentration and Self-Control	40 × 45 min sessions (5 months)	Attention, working memory, and impulsive behaviors	Stop-signal test (Walk–No Walk Test), The Working Memory Dual Request Selective Task, Matching Figures MF-14, IPPDAI, IPPDAG
Shuai et al. (2017)	China	Clinical	CT	7–9	ODD; CD; Tics; Anxiety; Phobias	Passive	Healthy control group	44	88	Developed by the authors	36 × 45 min sessions (4 months)	Inhibition, working memory, planning, organization, shifting, theory of mind, time management, emotional regulation	Stroop Task, Rey-Osterrieth Complex Figure Test, TMT, BRIEF, ToH, ADHD Rating Scale-IV, CRS, false-belief task
Steiner et al. (2014)	USA	School	RCT	≈7–9 2nd to 4th grade	–	1 Passive1 Active	Treatment as usual; neurofeedback	34	36 + 34	BrainTrain website	40 × 45 min sessions (5 months)	Attention, working memory	CRS, BRIEF, SKAMP, Behavioral Observation of Students in Schools (BOSS)
Steiner et al. (2011)	USA	School	RT	≈12–14 6th to 8th grade	–	1 Passive1 Active	Waitlist; neurofeedback	13	15 + 13	BrainTrain website	36 × 45 min sessions (4 months)	Attention, working memory	CRS, BRIEF, Behavior Assessment Scales for Children−2, Integrated Visual and Auditory CPT
Tamm et al. (2013)	USA	–	RT	7–15	Unclear	Passive	Waitlist	54	51	Pay Attention!	16 × 30 min sessions (8 weeks)	Sustained, selective, alternating, and divided attention	SNAP-IV, BASC-II, CGI, ATTC, BRIEF, TEA-Ch, WJ-III, D-KEFS, Quotient ADHD system
Tamm and Nakonezny (2015)	USA	–	RT	3–7	–	Passive	Waitlist	13	12	e.g., Highlight, memory card games	8 × 60 min sessions (8 weeks)	Attention, inhibition, memory, hand–eye coordination, balance, sensory awareness, listening skills, visual focusing	BRIEF, CGI, K-SADS-PL, SNAP-IV, NEPSY, CELF-IV
van der Donk et al. (2015)	Netherlands	School	RCT	8–12	LD; ODD	Active	Combined WM- and EF compensatory training	50	50	Cogmed Working Memory Training, Paying attention in class	25 × 45 min sessions (5 weeks)	Working memory	Creature Counting and Score!, Digit Span, Comprehension of Instruction and Word List Interference, Span Board, Six Part test BADS-C, BRIEF, CBCL, TRF,
van der Oord et al. (2014)	Netherlands	Home	RT	8–12	ODD	Passive	Waitlist	18	22	Braingame Brian	25 × 50 min sessions (5 weeks)	Visuospatial working memory, inhibition, and cognitive flexibility	BRIEF, DBDRS
van Dongen-Boomsma et al. (2014)	Netherlands	Home	RCT	5–7	ODD; Elimination disorder; PDDNOS; DCD; DBNOS	Passive	Placebo	26	21	Cogmed Working Memory Training	25 × 15 min sessions (5 weeks)	Working memory	ADHD Rating Scale-IV, BRIEF, Digit Span, Knox Cubes LDT, Sentences (WPPSI-R), RCPM, Day-Night Stroop task, SA-DOTS-02K, Shape School

*RCT, Randomized controlled trial; RT, Randomized trial; CT, Controlled trial; OP/OT, Open-label/Open-trial; ODD, Opposition Defiant Disorder; CD, Conduct Disorder; LD, Learning Disabilities; PDDNOS, Pervasive Developmental Disorder Not Otherwise Specified; DCD, Developmental Coordination Disorder; DBNOS, Disruptive Behavior Not Otherwise Specified*.

*CPT, Conners' Continuous Performance Test; TOL, Tower of London; RCPM, Raven's Colored Progressive Matrices; SNAP-IV, Swanson, Nolan and Pelham Questionnaire−4th Edition; BRIEF, Behavior Rating Inventory of Executive Functions; CRS, Conners' Rating Scale; CBCL, Child Behavior Checklist; TRF, Teacher Report Form; SDQ, Strengths and Difficulties Questionnaire; WFIRF, Weiss Functional Impairment Rating Scale; TMT, Trail Making Test; WCST, Wisconsin Card Sorting Test; IPDDAI, Early Identification of ADHD for Parents; IPDDAG, Early Identification of ADHD for Teachers; CANTAB, Cambridge Neuropsychological Test Automated Battery; CBTT, Corsi Block Tapping Test; DBDRS, Disruptive Behavior Disorders Rating Scale; RAST, Restricted Academic Setting Task; SSRS, Social Skills Rating System; WISC, Wechsler Intelligence Scale for Children; FAS, Phonological Fluency Test; CHEXI, Childhood Executive Functioning Inventory; ToH, Tower of Hanoi; SKAMP, Swanson, Kotkin, Agler, M-Flynn and Pelham Scale; CGI, Clinical Global Impressions; WJ-III, Woodcock-Johnson Tests of Achievement−3rd edition; D-KEFS, Delis-Kaplan Executive Functioning System; BASC-II, Behavioral Assessment System for Children−2nd edition; ATTC, Attentional Control Scale; TEA-ch, Test of Everyday Attention for Children; K-SADS-PL, Kiddie Schedule for Affective Disorders and Schizophrenia for school-aged children—Present and Lifetime Version; NEPSY, Developmental Neuropsychological Assessment Battery; CELF-IV, Clinical Evaluation of Language Fundamentals−4th edition; WPPSI-R, Wechsler Preschool and Primary Scale of Intelligence; SA-DOTS-02K, Sustained Attention Dots Task*.

Sample groups were composed by children diagnosed with ADHD and their typically developing peers. Twelve studies included samples of participants with no comorbidities and seven studies included children with comorbid diagnosis such as Opposition Defiant Disorder (ODD), Conduct Disorder (CD), Learning Disabilities (LD), Anxiety and/or Tics, Mood Disorders, Phobias and/or Elimination Disorders. Three studies did not detail whether they included or excluded participants with comorbidities (cf. Table 2).

Regarding research design, 10 studies were randomized controlled trials, eight were randomized trials, two were open-label/open-trial designs, and two were non-randomized controlled trials (cf. Table 2). In regard to control groups, three of these studies compared the performance of the experimental groups only with active control groups (i.e., medication, non-adaptive training, different intervention) and 14 included passive control groups (i.e., waitlist, healthy control group, normal school activities, treatment as usual) (cf. Table 2). Furthermore, to assess outcomes, eight studies included informants blind to study conditions (i.e., uninformed regarding which people belong to a particular group). One study included only blinded teachers (Beck et al., 2010) and three included blinded classroom observers (Green et al., 2012; Steiner et al., 2014; Minder et al., 2018). In three studies all informants were blind, either parents, teachers, and/or clinicians (van Dongen-Boomsma et al., 2014; Dovis et al., 2015; Bigorra et al., 2016). Lastly, one study used blind clinician assessments (Tamm and Nakonezny, 2015) and another blinded researchers and participants (Green et al., 2012). The remainder fourteen studies used unblinded informants.

Seven studies included in this review were carried out in the USA and 10 in Europe. Two studies were carried out in China, one in Brazil, one in Iran, and one in Australia (cf. Table 2). Eight interventions were conducted at home, six took place in school settings, and four in clinical settings. One study was carried out both in school and clinical settings (Minder et al., 2018). This information was not explicitly stated in three studies. When interventions took place at home, parents where usually the ones providing support during sessions. At school, the intervention was either delivered by teachers, clinicians, or trained research assistants. In regard to the clinical setting, most of the programs were employed by clinicians, but it was not possible to gather precise information in three of these investigations.

Nine of the 22 included studies were published between 2010 and 2014, while five studies were published in 2015, two in 2016, three in 2017, and three in 2018 (cf. Table 2).

### Type of Intervention

The duration of interventions varied extensively. The number of the training sessions varied from 8 to 64, and each session lasted from 15 min to 2 h (average of 52 min). The amount of time participants spent in training ranged between 375 and 3,840 min (average of 1,096 min per intervention).

Two types of intervention materials were considered: computerized (Beck et al., 2010; Steiner et al., 2011, 2014; Green et al., 2012; Johnstone et al., 2012; Egeland et al., 2013; van der Oord et al., 2014; van Dongen-Boomsma et al., 2014; Dovis et al., 2015; Azami et al., 2016; Bigorra et al., 2016; Davis et al., 2018; Minder et al., 2018) and non-computerized cognitive training (Tamm et al., 2013; Menezes et al., 2015; Re et al., 2015; Tamm and Nakonezny, 2015; Hannesdottir et al., 2017; Qian et al., 2017; Shuai et al., 2017; Capodieci et al., 2018). One study used both types of intervention in order to compare their efficacy (van der Donk et al., 2015).

Regarding the computerized training, the majority of the studies employed some variation of the Cogmed software. Additional websites and software encompass the Persian software of working memory training, Project: Evo, Braingame Brian, CogniPlus, the Brain Train website, and others. Further programs and activities, without computerized characteristics, feature the activities presented in the manual published by Caponi and collaborators, the OutSMARTers program, PIAFEx, a training program based on Dawson and Guare's training of executive skills for children, and other activities. One study did not specify the training program used during the intervention but provided some insights about its characteristics (Shuai et al., 2017).

### Targets of Intervention

As previously stated, there is no universally accepted definition of EF and, therefore, there is no agreement concerning the elements incorporated in this construct. Consequently, there is extensive variability in the domains of intervention across studies, and, in total, 16 EF were considered. Five of these 16 domains were targeted more frequently, specifically: attention (*n* = 8), inhibition (*n* = 8), working memory (*n* = 16), planning (*n* = 3), and cognitive flexibility (*n* = 4). Other components of executive functioning addressed by interventions incorporate organization, processing speed, short-term memory, self/emotional regulation, time management, and theory of mind (cf. Table 2).

### Outcome Measures

A variety of measures were used across studies to measure EF, as detailed in Table 3. These measures can be subdivided into two categories: (1) performance-based neuropsychological measures (i.e., computer-oriented or pen-paper tasks) and (2) behavioral and EF questionnaires (under the perspective of parents, teachers, clinicians and/or significant others).

**Table 3:** Performance-based measures and questionnaires employed across studies.

**Performance-based measures of attention and EF**	
Conners Continuous Performance Test	Knox Cubes LDT
Tower of London	Cancellation Attention Test
Digit Span	Phonological Fluency Test
Raven Colored Progressive Matrices	Animals Verbal Fluency Test
Span Board	Developmental Neuropsychological Battery
Trail Making Test	Clinical Evaluation of Language Fundamentals−4th edition
Auditory Working Memory Test	Walk/No-Walk Test
Visual Working Memory Test	Selective Working Memory Test
Wisconsin Card Sorting Task	Matching Figures Test (MF-14)
Spatial Span	Visual Go/No-Go Task
Cambridge Neuropsychological Test Automated Battery	Oddball Task
Test of Variables of Attention	Flanker Task
Stop Task	Counting Span
Tower of Hanoi	The Working Memory Dual Request Selective Task
Woodcock-Johnson Tests of Achievement−3rd edition	Letter-number Sequencing (WISC-III/IV)
Stroop Test	Coding (WISC-IV)
Corsi Block Tapping Test	Arithmetic (WISC-IV)
Delis-Kaplan Executive Functioning System	Creature Counting and Score!
Rey-Osterrieth Complex Figure	Comprehension of Instruction and Word List Interference
Test of Everyday Attention for Children	Sentences (WPPSI-RN)
Quotient ADHD System	Sustained Attention Dots Task
Shape School	Behavioral Assessment of the Dysexecutive Syndrome in Children
**Ratings of symptomatology and EF**	
Swanson, Nolan and Pelham Questionnaire−4th Edition	Behavior Assessment Scales for Children−2
Behavior Rating Inventory of Executive Function	Clinical Global Impressions
Conners Rating Scales	Childhood Executive Functioning Inventory
Child Behavior Checklist	Attentional Control Scale
Teacher Report Form	Swanson, Kotkin, Agler, M-Flynn and Pelham Scale
Strengths and Difficulties Questionnaire	IPDDAI (Early Identification of ADHD for Parents)
WEISS Functional Impairment Rating Scale	IPDDAG (Early Identification of ADHD for Teachers)
Disruptive Behavior Disorders Rating Scale	Kidscreen-27
ADHD Rating Scale IV	Kiddie Schedule for Affective Disorders and Schizophrenia for school-aged children
Emotion Regulation Checklist	Social Skills Rating System

Within the 22 studies, 44 performance-based measures were used to assess particular aspects of EF. Of these 44 measures, 10 were adopted in three or more studies, respectively: (1) Conners' Continuous Performance Test, (2) Trail Making Test, (3) Wisconsin Card Sorting Task, (4) Delis-Kaplan Executive Functioning System, (5) Stroop Test, (6) Rey-Osterrieth Complex Figure, (7) Digit Span, (8) Raven Colored Progressive Matrices, (9) Developmental Neuropsychological Assessment Battery, and (10) Letter-Number Sequencing. It is important to note, however, that not all studies included in this review used performance-based measures to verify the efficacy of their intervention.

Furthermore, within the 20 ratings employed, five were widely used across studies, specifically: (1) Behavior Rating Inventory of Executive Functions, (2) Conners' Rating Scales, (3) Swanson, Nolan, and Pelham Questionnaire−4th edition, (4) Strengths and Difficulties Questionnaire, and (5) ADHD Rating Scale-IV.

### Effects of Intervention

Data for EF outcomes were examined in each study in order to determine the efficacy of the interventions, and details are outlined in Table 4. Of the 22 studies included in this review, 14 reported improvements in performance-based measures of EF (Green et al., 2012; Johnstone et al., 2012; Egeland et al., 2013; Tamm et al., 2013; Dovis et al., 2015; Menezes et al., 2015; Re et al., 2015; van der Donk et al., 2015; Azami et al., 2016; Bigorra et al., 2016; Shuai et al., 2017; Capodieci et al., 2018; Davis et al., 2018), four didn't resort to these measures to evaluate the outcomes (Beck et al., 2010; Steiner et al., 2014; van der Oord et al., 2014; Minder et al., 2018), four were unable to find significant differences between groups (van Dongen-Boomsma et al., 2014; Tamm and Nakonezny, 2015; Hannesdottir et al., 2017; Qian et al., 2017), and one was unclear regarding its findings (Steiner et al., 2011). In the 14 studies that presented positive results in performance-based measures, improvements were reported for attention (Johnstone et al., 2012; Tamm et al., 2013; van der Donk et al., 2015; Azami et al., 2016; Bigorra et al., 2016; Davis et al., 2018), working memory (Green et al., 2012; Dovis et al., 2015; Menezes et al., 2015; Re et al., 2015; van der Donk et al., 2015; Shuai et al., 2017; Capodieci et al., 2018; Davis et al., 2018), inhibition (Dovis et al., 2015; Menezes et al., 2015; van der Donk et al., 2015; Azami et al., 2016; Bigorra et al., 2016; Davis et al., 2018), visuospatial short-term memory (Dovis et al., 2015; Azami et al., 2016), verbal short-term memory (Azami et al., 2016), attentional control (Re et al., 2015; Capodieci et al., 2018), interference control (Dovis et al., 2015; Shuai et al., 2017), impulsiveness (Re et al., 2015; Capodieci et al., 2018), processing speed (Egeland et al., 2013; Shuai et al., 2017), shifting (Shuai et al., 2017), planning (Tamm et al., 2013; Shuai et al., 2017), and reasoning (Azami et al., 2016). The effect sizes reported ranged from small to large (cf. Table 4 for detailed results).

**Table 4:** Results of included studies.

	**Study**	**Effect sizes**		**Main findings**
1	Azami et al. (2016)	CPT (total correct): *d* = 1.12 Raven's progressive matrices: *d* = 1.436 Backward digit span: *r* = −0.721(placebo); *r* = −0.11 (medication) SNAP-IV (ADHD-PHI): *d* = 1.784	Span board: *d* = 1.34 Forward digit span: *r* = −0.567 (placebo); *r* = −0.037 (medication) SNAP-IV (ADHD-C): *d* = 1.422	For simple EF tasks (e.g., sustained attention and inhibition), the experimental group had the same results as the active stimulant medication group. However, for a number of complex EFs (e.g., verbal and visuospatial short-term memory and non-verbal reasoning), the experimental group showed better results than the active stimulant medication and placebo groups.
2	Beck et al. (2010)	ADHD index: *d* = 0.76 Cognitive problems/inattention: *d* = 0.79 Hyperactivity: *d* = 0.36 DSM-IV inattentive scale: *d* = 1.49 BRIEF Teacher Scale Initiate: *d* = 0.42	BRIEF Parent Scale Metacognition index: *d* = 0.91 Working memory: *d* = 0.85 Initiate: *d* = 0.94 Plan|organize: *d* = 0.92	The experimental group showed better results on parent ratings of overall ADHD symptoms, inattention, initiation, planning/organization, and working memory than the waitlist control group.
3	Bigorra et al. (2016)	Working memory composite score: *d* = 0.81 CPT (detectability): *d* = 0.60 BRIEF Parent Scale Working memory: *d* = −0.86 Plan|organize: *d* = −0.71 Metacognition index: *d* = −0.78 ADHD symptom composite Parents: *d* = −0.39 Teachers: *d* = −0.69	CPT (commission errors): *d* = 0.40 BRIEF Teacher Scale Initiate: *d* = −0.55 Working memory: *d* = −0.36 Monitor: *d* = −0.72 Shift: *d* = −0.39 Metacognition: *d* = −0.37 School learning behavior (WFIRS-P): *d* = −0.86	The experimental group improved significantly more than the control group on parent ratings of the metacognition index (i.e., the child's ability to monitor, initiate, plan, organize, and sustain future-oriented problem solving in working memory). Also, the experimental group improved significantly more than the control group on teacher ratings of the metacognitive index, initiation, working memory, monitoring, and shifting. Also, for the experimental group compared to the control group there were significant improvements in performance-based measures of EF, ADHD symptoms, and functional impairment.
4	Capodieci et al. (2018)	Forward digit span: *d* = 0.72 Backward digit span: *d* = 1.70 Selective working memory: *d* = 1.70	Walk-No walk: *d* = 1.25 MF-14: *d* = 1.29	The experimental group showed better results than the control group in performance-based measures of working memory and other neuropsychological measures. Effects were not found for inattention and hyperactivity problems rated by teachers and parents.
5	Davis et al. (2018)	Test of Variables of Attention Attention Performance Index: *d* = 0.35 Attention Performance Index (high severity): *d* = 0.71	Reaction Time Mean (high severity): *d* = 0.65 Reaction Time Variability (high severity): *d* = 0.62	The experimental group showed more improvements than the control group on performance-based measures of attention, working memory, and inhibition than the control group, especially among children with greater symptom severity and impaired attention.
6	Dovis et al. (2015)	Corsi Block Tapping Test Forward: $\eta_{p}^{2}=$ 0.16 Backward: $\eta_{p}^{2}=$ 0.09		Only children in the full-active condition (where working memory, inhibition, and cognitive flexibility were trained) compared to a partially-active condition (where only inhibition and cognitive flexibility were trained) and to a placebo condition showed better results on measures of visuospatial short-term memory and working memory.
7	Egeland et al. (2013)	CPT (Processing speed): η^2^ = 0.105		The experimental group presented better results than the control group only in processing speed. Reading and mathematics were improved in the experimental group, changes in ADHD symptom rating scales were not visible. In addition, the improvements in reading scores remained significant 8 months later.
8	Green et al. (2012)	—		The experimental group presented reductions in off-task ADHD-associated behaviors after training. Improvements in working memory performance-based measures were also found. No significant improvements were found on parent rating scales.
9	Hannesdottir et al. (2017)	ADHD-RS-IV (Parent) Inattention: *d* = 0.90 Hyperactivity|Impulsivity: *d* = 0.74 Strengths and Difficulties Questionnaire Total score: *d* = 0.75	Social Skills Rating System Total score: *d* = 0.54 Emotion Regulation Checklist Emotion regulation: *d* = 0.67	Compared to a waitlist control group, the experimental group (OutSMARTers Program) showed a reduction of ADHD symptomatology, improved social skills and better emotion regulation according to parents. No improvements were found on performance-based measures. No differences were found between the experimental and a parent training group, as both groups showed some improvement. These improvements were still visible 3 months later.
10	Johnstone et al. (2012)	—		Children in both experimental groups (i.e., working memory and inhibitory control training with and without attention monitoring) showed significant improvements in ratings of ADHD symptomatology according to parents and other family members. Better performance in tasks pertaining to spatial working memory, ignoring distracting stimuli, and sustained attention were also reported, with the attention monitoring via EEG retaining little effect on the outcomes. The follow-up sessions carried after a 6-week interval revealed maintenance of gains.
11	Menezes et al. (2015)	—		The experimental group showed better performance on measures of attention/inhibition and auditory working memory compared to the control group. No effect was found for measures of more complex executive functions, such as flexibility, visual working memory, and verbal fluency. Parent rating scales showed no improvement of ADHD symptomatology or executive functioning.
12	Minder et al. (2018)	Conners-3 ADHD DSM-IV indices (Parent) Inattention: η^2^ = 0.096		Both experimental groups (cognitive training vs. neurofeedback) improved in ratings of ADHD symptomatology and executive functions according to parents and teachers and off-task behavior as reported by blinded raters. An effect of training was found for cognitive training only on inattention symptoms rated by parents.
13	Qian et al. (2017)	—		After the intervention, children in the experimental group were rated by parents as displaying improved executive functioning, diminished ADHD symptomatology, reduced risk-tasking behaviors and enhanced academic performance. Despite these improvements, the experimental group was still distinguishable from the healthy control group in almost all variables.
14	Re et al. (2015)	Walk-No walk: $\eta_{p}^{2}$ = 0.27	MF-14 (errors): $\eta_{p}^{2}$ = 0.37	Children with ADHD presented better performance in tasks assessing attention, inhibition, and working memory. Improvements in children with typical development who attended the training were also found. Both parents and teachers' ratings of ADHD symptomatology improved for the experimental and control groups.
15	Shuai et al. (2017)	—		The experimental group presented better performance in neuropsychological tests after the intervention, with improvements in processing speed, inhibition, shifting, working memory, and planning. Results from parent rating scales showed reduced ADHD symptomatology and behavioral problems as well as improved executive functioning and academic performance. At post-test, there were no significant differences between the ADHD and healthy control groups.
16	Steiner et al. (2014)	—		Only children who attended the neurofeedback intervention showed significant improvement in ratings of attention, executive functioning and off-task behavior compared with those in the control and cognitive training conditions.
17	Steiner et al. (2011)	Conners Rating Scales-Revised Inattention: *d* = 0.80 ADHD index: *d* = −0.70	Behavior Assessment Scales for Children −2 Attention Problems: *d* = −0.80 Behavior Rating Inventory of Executive Function Global Executive Composite: *d* = −0.60	The experimental group that received Neurofeedback training was rated by parents as presenting less symptoms of ADHD and improved behavior. Parents of children that attended the Standard Computer Format training reported less inattention and ADHD symptoms as well as improvements in executive functioning. Teacher and self-report ratings did not show symptomatology improvements.
18	Tamm et al. (2013)	SNAP-IV (Parent) Inattention: *d* = 1.65 Hyperactivity|Impulsivity: *d* = 0.65 SNAP-IV (Clinician) Inattention: *d* = 1.41 Hyperactivity|Impulsivity: *d* = 0.68 Behavior Assessment Scales for Children−2 Attention Problems (parent): *d* = 0.66 Clinical Global Impressions Severity: *d* = 1.04 Improvement: *d* = 1.14	D-KEFS Tower time per move ratio: *d* = 0.55 BRIEF Parent Scale Shift: *d* = 0.63 Initiate: *d* = 0.98 Working memory: *d* = 1.16 Planning: *d* = 1.00 Organization: *d* = 0.53 Monitor: *d* = 0.70 Behavioral Regulation Index: *d* = 0.63 Metacognition Index: *d* = 1.13 General Executive Composite: *d* = 1.03	After the intervention, the experimental group performed significantly better on a measure of planning compared to a waitlist control group. No effects were found in the remainder performance-based measures. Parents rated children in the experimental group as presenting fewer ADHD symptoms and better executive functioning. Clinician ratings of ADHD symptoms presented reduced scores and children reported a better ability to focus and shift attention. Teacher ratings did not reach statistical significance.
19	Tamm and Nakonezny (2015)	BRIEF Parent Scale Shift: *d* = 1.01 Emotion Regulation: *d* = 0.97	SNAP-IV (Clinician) Inattention: *d* = 1.10	No improvements were found in performance-based measures of executive functions for the experimental group compared to the waitlist control group following the intervention. However, parents of children in the experimental group reported effects on the shift and emotion regulation subscales of the BRIEF. Blinded clinicians' ratings revealed decreased inattention symptoms.
20	van der Donk et al. (2015)	Creature counting (correct): *d* = 0.26 Word list interference (remember): *d* = −0.33 BRIEF Teacher Scale Metacognition Index: *d* = −0.07	Span board: *d* = 0.85 Comprehension of instructions: *d* = −0.08 BRIEF Parent Scale Behavioral Regulation Index: *d* = −0.05 Metacognition Index: *d* = 0.01	Both experimental groups (Cogmed working memory training vs. Pay Attention in Class) improved on measures of attention, inhibition, and planning. Parent and teacher ratings of executing functioning and ADHD symptomatology presented decreased scores, but no effects were found on academic, behavioral, and quality of life outcomes.
21	van der Oord et al. (2014)	Disruptive Behavior Disorder Rating Scale Inattention: η^2^ = 0.25 Hyperactivity|Impulsivity: η^2^ = 0.22	BRIEF Parent Scale Metacognition Index: η^2^ = 0.16 Global Executive Composite: η^2^ = 0.16	The experimental group showed better results on parent ratings of ADHD symptomatology as well as on the metacognition index and total score of the BRIEF compared to a waitlist control group. These effects maintained stable at the 9-week follow-up and improvements on inhibition were found. Teacher ratings showed no effects of training at post-test but revealed improvements from pre-test to follow-up on ADHD symptomatology.
22	van Dongen-Boomsma et al. (2014)	—		No significant treatment effect was found on the outcome measures applied. The experimental and placebo groups did not differ at the end of the intervention on behavioral symptoms, neurocognitive performance, executive and global functioning.

Regarding the ability of cognitive training to trigger change in day-to-day life (i.e., transfer effects; Toplak et al., 2008), 11 studies have shown decreases in parent and/or teacher ratings of ADHD symptomatology and 11 studies conveyed reductions on EF difficulties according to informants, with small to large effect sizes (cf. Table 4). Studies also stated improvements in social skills (Hannesdottir et al., 2017; Qian et al., 2017), progress in academic performance (Egeland et al., 2013; Qian et al., 2017; Shuai et al., 2017), and reduced off-task behavior (Green et al., 2012; Minder et al., 2018; cf. Table 4). In the matter of the assessment of EF behaviors in everyday environments, informants reported positive changes in working memory (Beck et al., 2010; Tamm et al., 2013; Tamm and Nakonezny, 2015; Bigorra et al., 2016; Shuai et al., 2017), initiation (Beck et al., 2010; Tamm et al., 2013; Shuai et al., 2017), planning/organization (Beck et al., 2010; Tamm et al., 2013; Tamm and Nakonezny, 2015; Shuai et al., 2017), monitoring (Tamm et al., 2013; Bigorra et al., 2016; Shuai et al., 2017), shifting (Tamm et al., 2013; Bigorra et al., 2016), inhibition (Tamm and Nakonezny, 2015; Shuai et al., 2017), emotional control (Shuai et al., 2017), the metacognition (Tamm et al., 2013; van der Oord et al., 2014; van der Donk et al., 2015; Bigorra et al., 2016; Shuai et al., 2017; Minder et al., 2018) and behavioral regulation indexes (Tamm et al., 2013; van der Donk et al., 2015; Shuai et al., 2017; Minder et al., 2018), and the global executive composite (Steiner et al., 2011; Tamm et al., 2013; van der Oord et al., 2014; Shuai et al., 2017).

Of these 22 studies, 13 did not analyze the possible maintenance of gains through follow-up sessions. Of the nine studies that performed follow-up sessions, seven concluded that the gains observed at the end of the intervention were maintained throughout time (Beck et al., 2010; Johnstone et al., 2012; Egeland et al., 2013; van der Oord et al., 2014; van der Donk et al., 2015; Bigorra et al., 2016; Hannesdottir et al., 2017).

In order to assess if study design had implications in the results obtained across studies, a qualitative comparison of the results obtained in randomized (*n* = 18) vs. non-randomized (*n* = 4) trials was conducted. On one hand, randomized studies reported, more often than non-randomized trials, improvements in ratings (≃33 vs. ≃25%) as well as in performance-based measures and ratings combined (≃33 vs. ≃25%). On the other hand, non-randomized trials reported improvements only in performance-based measures more frequently than randomized studies (≃50 vs. ≃22%).

As previously reported, seven studies contemplated in this review included samples of children with comorbid diagnoses. A qualitative comparison of the results obtained by these studies showed that, comparatively to studies that included children with an ADHD diagnosis only, a higher proportion of studies with included comorbid diagnoses reached improvements in ratings (≃43 vs. ≃25%) and performance-based measures in combination with ratings (≃43 vs. ≃25%). Also, in samples without comorbid diagnoses, improvements were found more frequently only in performance-based measures (≃42%).

## Discussion

The first aim of this study was to update and extend the findings of previous reviews, characterizing the current literature on cognitive training interventions for EF in children and adolescents diagnosed with ADHD between 3 and 14 years of age. A total of 741 articles were identified and, after duplicates removal, 517 articles were analyzed. Twenty-two studies were eligible for inclusion. Regarding the characteristics of the included studies, it is useful to highlight important methodological features. Studies tend to include individuals diagnosed with ADHD and other comorbid disorders (i.e., ODD, CD, LD, Anxiety and/or Tics, Mood Disorders, Phobias, and/or Elimination Disorders). Regarding research designs, the majority of the included studies were randomized trials, but a few non-randomized studies were also included as they fulfilled the pre-established inclusion criteria. Also, although all studies included control groups, unblinded outcomes assessments were performed frequently. Most of the research was conducted in USA and Europe in home, school, and/or clinical settings. Regarding intervention programs, computerized but also non-computerized programs were frequently employed. The most frequent EF domains targeted by interventions were attention, inhibition, working memory, planning, and cognitive flexibility.

Our second aim was to assess whether cognitive training was effective in ADHD as evaluated by performance-based measures of EF and/or behavioral/EF questionnaires. Results showed that most of the studies that used performance-based measures demonstrated efficacy in improving one or more domains of EF in children and adolescents. Study design appears to have implications in the results obtained across studies, as a qualitative comparison of the results obtained in randomized and non-randomized trials showed that randomized studies reported, more often than non-randomized trials, improvements in ratings as well as in performance-based measures and ratings combined. Conversely, non-randomized trials reported improvements only in performance-based measures more frequently than randomized studies.

Furthermore, we intended to evaluate the transfer of gains (i.e., the generalization of training effects to other non-trained tasks). Regarding these transfer effects, research has shown decreases in parent and/or teacher ratings of ADHD symptomatology, social skills improvements, and reductions in EF dysfunction in daily life. Additionally, improvements in academic performance and reduced off-task behavior (i.e., task disengagement to engage in unrelated behaviors) were reported. In spite of these results showing that cognitive training can be an effective intervention for children and adolescents with ADHD, our conclusions should be interpreted considering limitations of the included studies as discussed below.

As previously mentioned, several studies integrated in this review included participants with associated comorbidities. In fact, Efron et al. (2016) found that in a sample of 132 diagnosed children, aged 4–7 years, 39% had one comorbidity and 37% had more than one comorbidity. According to the authors, ODD (53%), Anxiety Disorder (23.5%), LD (15.9%), and Language Disorder (14.4%) were the most common comorbidities across individuals with ADHD. Similar results have been reported by Reale et al. (2017). The authors found that of the 1,919 subjects evaluated, 66% had at least one comorbid diagnosis, while only 34% presented just ADHD. Among the most common comorbid diagnosis were LD (56%), Sleep Disorders (23%), ODD (20%), and Anxiety Disorders (12%). Therefore, it is clear that the majority of children diagnosed with ADHD have, at least, one comorbid disorder.

The comparison of the results obtained by the included studies in this review demonstrated that a higher proportion of studies that included comorbid diagnoses reached improvements in ratings and performance-based measures in combination with ratings. Contrarily, in samples without comorbid diagnoses, improvements were found more frequently only in performance-based measures. These results suggest that, for children with comorbidities, improvements perceived by informants in daily life were more frequent. In fact, as discussed by Diamond (2012), children with greater difficulties on executive functioning may benefit the most from any intervention focusing on its training. As children with comorbid diagnoses usually present with higher levels of symptomatology and EF problems, they may have more room for improvement than children presenting with one single diagnosis (Flook et al., 2010). As such, individuals with different comorbidities may respond differently to specific treatments, have differing clinical correlates, and/or demonstrate unique clinical outcomes what might influence results obtained across studies. So, additional studies should group ADHD individuals into more homogenous subgroups based on comorbid patterns.

Regarding control groups, in order to consider that differences between the groups may be accounted to effects of the intervention, researchers should compare the performance of an experimental group and a control group that “accounts for improvements caused by factors other than the treatment” (i.e., an active control group; Boot et al., 2013). In line with these theoretical assumptions, assessing the efficacy of interventions by comparing the performance between a treated group and a non-treated group (i.e., treatment as usual, waitlist, typical development) would not be appropriate since both groups have different expectations (Boot et al., 2013), influencing results on outcome measurements. In fact, of the studies included in this review, three reported active control groups and five used both active and passive control groups. Nevertheless, the majority of the comparison groups were passive (*n* = 14), and these considerations should be taken into account while interpreting these results.

Another limitation of most of the studies conducted is the lack of transfer effects when the efficacy of the intervention is assessed by blinded raters. Even though this was confirmed across the majority of studies that included unblinded informants, positive results should also be highlighted. Beck et al. (2010) and Bigorra et al. (2016) assessed treatment efficacy through blinded raters and found significant results. Beck et al. (2010) found slight improvements in the initiate scale of the BRIEF—Teacher Form, even though these only approached significance. Bigorra et al. (2016) found significant improvements in several subscales of the BRIEF—Teacher and Parent Forms, with small to large effect sizes. The authors also found significant improvements in ADHD symptoms according to both teachers and parents. These results show that blind raters are able to detect changes in everyday situations following cognitive training interventions. Nevertheless, more studies using blind raters are needed in order to provide a better understanding of the effects of cognitive training.

For future studies, there are a number of additional issues that researchers must address to support empirical evidence for the implementation of EF training as a complementary intervention for individuals with ADHD. Specifically, a well-designed intervention should (a) randomize participants into the experimental and control groups; (b) match participants in variables that might account for differences between groups other than the treatment (e.g., age, comorbidity); (c) control for participants and informants expectations through blinding and assessment of expectations prior to the beginning of the intervention to control for possible placebo effects; (d) compare the performance of the experimental group to both active and passive control groups; and (e) use both performance-based measures and ratings of EF and behavior to assess the interventions' efficacy.

Notwithstanding, this review represents an important contribution as it includes a wider range of studies (i.e., different designs and interventions), having important clinical and educational implications, as it demonstrates the feasibility and positive effects of conducting EF training with children and adolescents with ADHD in a variety of contexts.

In sum, our results showed that cognitive training can be an effective intervention for children and adolescents with ADHD and might be considered a complement of psychostimulant medication. Nonetheless, conclusions should be interpreted with caution due to important methodological limitations. However, the available evidence certainly justifies the allocation of resources to evaluate the efficacy of EF interventions, since they carry the promise of reducing ADHD symptomatology and improving academic, interpersonal, and occupational outcomes.

## Author Contributions

AV and MF contributed to the conception and design of the work. AV prepared the first draft of the manuscript. MF revised the manuscript critically for important intellectual content. MF and SV revised the last version of the manuscript.

### Conflict of Interest

The authors declare that the research was conducted in the absence of any commercial or financial relationships that could be construed as a potential conflict of interest.
